# Oxidative Stress, Vascular Endothelium, and the Pathology of Neurodegeneration in Retina

**DOI:** 10.3390/antiox11030543

**Published:** 2022-03-12

**Authors:** Xin Shi, Panpan Li, Hanhan Liu, Verena Prokosch

**Affiliations:** Department of Ophthalmology, Faculty of Medicine and University Hospital of Cologne, University of Cologne, 50937 Cologne, Germany; shixinsx123@gmail.com (X.S.); panpanlimed@gmail.com (P.L.); hanhan.liu@uk-koeln.de (H.L.)

**Keywords:** oxidative stress, endothelium, neurodegeneration, retina

## Abstract

Oxidative stress (OS) is an imbalance between free radicals/ROS and antioxidants, which evokes a biological response and is an important risk factor for diseases, in both the cardiovascular system and central nervous system (CNS). The underlying mechanisms driving pathophysiological complications that arise from OS remain largely unclear. The vascular endothelium is emerging as a primary target of excessive glucocorticoid and catecholamine action. Endothelial dysfunction (ED) has been implicated to play a crucial role in the development of neurodegeneration in the CNS. The retina is known as an extension of the CNS. Stress and endothelium dysfunction are suspected to be interlinked and associated with neurodegenerative diseases in the retina as well. In this narrative review, we explore the role of OS-led ED in the retina by focusing on mechanistic links between OS and ED, ED in the pathophysiology of different retinal neurodegenerative conditions, and how a better understanding of the role of endothelial function could lead to new therapeutic approaches for neurodegenerative diseases in the retina.

## 1. Introduction

Oxygen, which plays a crucial role for living organisms, is also considered a double-edged sword. It can be actively involved in signal transduction, gene transcription, and multiple cellular activities, but it also generates harmful effects on biomolecules in the form of free radicals. Highly reactive atoms or molecules with one or more unpaired electrons in their outer shells can form radicals when oxygen interacts with certain molecules [1], such as the hydroxyl radicals (HO•), superoxide radical anion (•O_2_^•^), hydroperoxyl radicals (HO_2_•), and peroxyl radicals (ROO•). Free radicals can behave as oxidants or reducers in cells by accepting or losing an electron [2]. Reactive oxygen species (ROS) and reactive nitrogen species (RNS) refer to reactive free radicals and nonradical derivatives of oxygen and nitrogen, respectively. ROS/RNS can be produced by all aerobic cells [3]. “ROS” stands for both oxygen free radicals and nonradicals (H_2_O_2_, 1O_2_, etc.) that can be conveniently converted to free radicals [4,5,6,7]. ROS/RNS are not only associated with energy extraction, immune defense, and signaling processes but may also generate harmful effects [8]. Under normal circumstances, homeostasis ROS act as secondary messengers in various intracellular signaling pathways of the cardiovascular system [9]. Nevertheless, once the production of ROS/RNS and other oxidants exceeds the antioxidant defense, they may trigger cellular oxidative stress (OS) [10]. OS may contribute to subsequent oxidative modifications or damage to lipids, proteins, and DNA, with deleterious consequences for metabolism and cardiovascular disease [9], which is considered to be responsible for the pathogenesis of numerous age-related neurodegenerative diseases. ROS contribute significantly to the degeneration of neuronal cells by regulating the function of biomolecules. ROS involved in neurodegeneration includes hydrogen peroxide (H_2_O_2_), superoxide anions (O_2_^•^), and highly reactive HO•. O_2_^•^ is generated by adding an electron to oxygen, and several mechanisms exist to generate superoxide in vivo [11]. Moreover, electron transport chains in the inner membrane of mitochondria reduce oxygen to water. During this process, free radical intermediates are produced, which are usually tightly bound to the components of the transport chain. However, some electrons keep leaking into the mitochondrial matrix, which leads to the formation of superoxide [12,13]. In addition, the vascular endothelium probably produces O_2_^•^ continuously to neutralize nitric oxide, other cells generate superoxide to regulate cell growth and differentiation, and phagocytes produce superoxide during oxidative bursts [14,15]. The results of a systematic review and meta-analysis of randomized controlled trials indicated that markers of oxidative stress were increased in glaucoma overall (effect size = 1. 64; 95% CI 1.20–2.09), which ranged from an effect size of 1.29 (95% CI 0.84–1.74) in serum to 2.62 (95% CI 1.60–3.65) in aqueous humor. Although antioxidative stress markers in serum were decreased (effect size = −0.41; 95% CI −0.72 to −0.11), several were increased in the aqueous humor (superoxide dismutase, effect size = 3.53; 95% CI 1.20–5.85; glutathione peroxidase, effect size = 6.60; 95% CI 3.88–9.31). In addition, the increase in some antioxidant markers is probably a protective response of the eye to oxidative stress [16].

Endothelial cells (ECs) form the innermost lining of the vasculature. In the retina, ECs tightly interact with other cells within the neurovascular system to regulate blood–retina barrier integrity, neurovascular coupling, immune signaling, and neuronal metabolism [17,18,19,20,21]. While endothelial function plays a key role in the homeostasis of retinal neurons, the endothelial function can be impacted by increased OS [22,23,24]. Special receptors on EC membranes initiate intracellular signal cascades in response to agonists that activate specific receptors or changes in cell surface shear stress caused by changes in blood flow rate. Gap junctions allow crosstalk between adjacent ECs, thereby allowing the transmission of intracellular reactions. Once activated, these cascades trigger the release of potent vasodilator substances, such as nitric oxide (NO) and prostaglandins and vasoconstrictors, such as endothelin and endothelium-derived vasoconstrictors [25,26,27]. At low concentrations, ROS act as vasodilators, but at high concentrations, it may result in vascular dysfunction [28]. Systemic oxidative stress is associated with reduced ocular hemodynamic flow [29], which is linked to vascular permeability. Significantly higher endothelin-1 blood levels have been found in patients with POAG [30]. The association of nitric oxide with glaucoma has been previously reported [31]. Therefore, this review focuses on the impact of OS on endothelial function and the roles of endothelial dysfunction (ED) in retinal neurodegenerative diseases.

## 2. The Role of Endothelial Function in Human Diseases

Endothelial cells are located in the intimal layer of the vascular wall, making up about 1.5% of the total body weight [32,33,34]. Over the years, the endothelium has been recognized as more than a simple barrier to boundary the vascular wall [32,35]. As the innermost layer of blood vessels, the endothelium is considered to be a dynamic, adaptive interface between the blood circulation and the internal environment. The vascular endothelial cells are subjected to hemodynamic forces [36]. At the same time, ECs also send out various necessary signals and regulate vascular permeability by producing many messengers, which have good biological functions [37,38]. The biomechanical signals from blood and surrounding tissues provide selective barriers for the permeability of macromolecular substances while separating blood components from vascular wall matrix and tissues. Depending on the needs of the tissue, endothelium regulates vascular tension and changes vascular diameter by releasing vasodilator molecules, such as prostacyclin (PGI2) and vasoconstrictor molecules, such as endothelin and thromboxane A2 [39]. In addition, ECs also regulate blood fluidity by secreting anticoagulants, procoagulants, and fibrinolytic substances, control angiogenesis by producing angiogenic growth factors, and regulate acute and chronic by expression of cytokines, chemokines, and adhesion molecules. The endothelium responds to hemodynamics through mechanical sensors [40]. Moreover, ECs can also reduce intracellular apoptosis by integrating neurohumoral and inducing adaptive signaling pathways [41]. On all counts, the endothelium has anti-inflammatory and anticoagulant effects, contributes to the anti-thrombus formation, and controls permeability, endothelium and muscle growth (reshaping blood vessel), and vasoconstriction.

## 3. Endothelium Dysfunction

The endothelium plays a significant role in regulating vascular tone through the synthesis and release of a range of endothelium-derived diastolic factors, including the vasodilators prostaglandins, NO, and endothelium-dependent hyperpolarizing (EDH) factors, as well as endothelium-derived contractile factors [42,43]. Endothelium dysfunction is defined as an imbalance in the production of vasodilators and vasoconstrictors, which predisposes the vascular system to a prothrombotic and proatherogenic phenotype [44]. ED is primarily caused by reduced production or weakened activity of endothelial-derived relaxing factors, which are associated with a multitude of diseases, including atherosclerosis, diabetes, coronary artery disease, hypertension, hypercholesterolemia, and hyperhomocysteinemia (HHcy) [45,46]. ED has a multitude of distinguishing features, including disruption of vascular tone, redox imbalance, increased inflammatory response within the vessel wall [47], vasoconstriction (due to the impaired activity of vasodilator mediators), overexpression of leukocyte and platelet adhesion molecules, platelet activation (the secretion of active substances, increased von Willebrand factor and tissue factor), mitosis, pro-oxidation, impaired coagulation (anticoagulant factors such as heparin decreased), atherosclerosis, and thrombosis [44,48,49,50].

**Figure 1:**
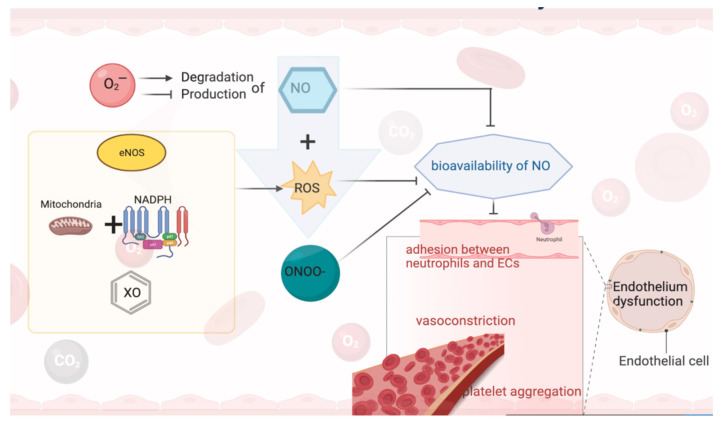
NO and endothelium dysfunction.

NO has a multi-biological regulatory role, including vascular smooth muscle cell relaxation and proliferation, leukocyte adhesion, angiogenesis, platelet aggregation, thrombosis, vascular tone, and hemodynamics, as well as inhibiting the production and/or activity of other vasoactive factors such as prostaglandins and endothelin-1 (ET-1) in response to vasoconstrictors [51]. When the NO-mediated reaction is functionally impaired, PGI2 and/or EDH contribute to functional compensation. Subsequently, as the ability of ECs to release NO gradually decreases, the production and activity of endothelium-derived cyclic oxygenase-dependent contractile factor (EDCF) and/or ET-1 becomes prominent, facilitating vasoconstriction. Simultaneously, due to the weakened protective effect of NO, the expression of endothelial cell adhesion molecules (e.g., vascular cell adhesion molecule 1 (VCAM-1), intercellular adhesion molecule 1 (ICAM-1)) is enhanced, which promotes leukocyte adhesion and infiltration. All these factors would result in the failure of the endothelium to perform its basic activities (i.e., regulation of vasodilation and/or cellular redox homeostasis), as well as decreased NO production is associated with damage to vascular smooth muscle dilation [52] (Figure 1).

## 4. Oxidative Stress and Endothelium Dysfunction

Oxidative stress (OS) occurs when there is an imbalance between ROS production and the antioxidant defense system [53]. NO is the primary endothelial regulator of local vascular tone, which is essential for maintaining vascular homeostasis in the endothelium. NO bioavailability decreased due to O_2_^•^-reducing NO production and/or increasing NO degradation, which marks the beginning of ED [54]. The free form of NO is hazardous to biomolecules. In addition, xanthine oxidase (XO), nicotinamide adenine dinucleotide phosphate (NADPH) oxidase, and uncoupled endothelial NO synthase (eNOS) as potential sources of enzymes may be responsible for increased ROS [55]. Mitochondria [56] react with membrane-related NADPH oxidase [57] to produce ROS. The increase in ROS production will dwindle the bioavailability of NO, thus promoting vasoconstriction, weakening the inhibition of platelet aggregation, as well as inducing the adhesion between neutrophils and ECs, while NO reacts with ROS to produce a strong oxidant—peroxynitrite (ONOO-). When NO reacts to peroxynitrite, NO no longer has normal physiological functions. These changes will provoke ED and perhaps cause alteration in intracellular signal pathways and transcription factor-mediated gene expression in ECs. OS caused by reduced NO production or increased ROS plays an important role in ED [58,59]. OS increases the phosphorylation of tyrosine kinases, such as focal adhesion kinase, post protein, and p130 cas in ECs, accompanied by increased stress fiber formation and neutrophil-endothelial cell adhesion [60,61]. Therefore, increased ROS and RNS levels and vascular OS is associated with ED [62,63]. The persistence of this condition probably induces endothelial contraction and death, increased permeability, and exposure of basement membrane components, which further amplifies the situation of vascular inflammation [48,62,64], causing significant damage to cells.

Vascular ROS act as significant intracellular signaling messengers, which could regulate vascular contractility, cell growth, and vascular remodeling [65]. Under normal physiological conditions, the majority of hazardous ROS are eliminated by the cellular antioxidant system. However, OS can trigger the pathogenesis of associated cardiovascular diseases [66]. Increased ROS production in blood vessels may result in pathologies associated with hypercholesterolemia, hypertension, diabetes, and aging [57]. It is well known that excess ROS contribute to lipid peroxidation and oxidative modifications of proteins and nucleic acids, which can cause ED [67]. OS and correlated oxidative damage are mediators of vascular damage and inflammation in numerous cardiovascular diseases, particularly in the setting of complications such as hypertension, hyperlipidemia, and diabetes [68,69]. The main source of OS in the arterial wall is NADPH oxidase (NOX) [70], which is involved in the production of ROS and the scavenging of NO [71]. In the endothelium, increased levels of ROS will reduce the bioavailability of NO, leading to vasoconstriction and ED. Other sources of ROS in the vascular wall include the mitochondrial respiratory chain and other enzymatic reactions, such as cyclooxygenases (COX), xanthine oxidase (XO), lipoxygenases (LOX), cytochrome P450, and dysfunctional eNOS [72,73,74]. Furthermore, the blood vessel wall is enriched with multiple enzymes, which could alleviate the ROS overload and act as an antioxidant defense system. These bio-enzymes include superoxide dismutase (SOD), catalase, glutathione peroxidase (GPx), hemoglobin oxygenase (HO), thioredoxin peroxidase (TPX), paraoxonase (PON) [72,75,76]. OS can be responsible for the oxidation of low-density lipoprotein (LDL), which inhibits the release of endothelium-derived NO or closely related molecules even more than natural or unoxidized LDL [24]. In addition, oxidized LDL (ox-LDL) exerts a multitude of biological effects, such as cytotoxicity to ECs, chemotaxis to monocytes, and the accumulation of inflammatory cells and ROS in the vascular system [77,78].

Interestingly, studies have confirmed that correcting hypercholesterolemia or treating cell cultures and experimental animals with SOD or SOD simulants can reduce vascular O_2_•-levels and restore endothelial function [79]. It has been reported that XO inhibitors hydroxypurinol or allopurinol can improve ED in hypercholesterolemia [80]. Therefore, XO may become a significant source of ROS production in vascular ECs and is related to ED in some vascular diseases. Moreover, the reduction of NO-dependent dilation induced by OS and alterations in vascular smooth muscle function directly contribute to microvascular dysfunction in major depressive disorder (MDD) [81].

La Favor et al. [82] exploited a novel microdialysis technique that allows simultaneous measurement of ROS levels and microvascular endothelial function in vivo. They discovered that elevated levels of NADPH oxidase-derived ROS in obese subjects were associated with microvascular ED, such as impaired acetylcholine-induced increases in blood flow. Interestingly, Gray et al. [83] demonstrated that NADPH oxidase 4-derived H_2_O_2_ provides vasoprotective effects in a mouse model of diabetic atherosclerosis. However, Gutterman’s laboratory has proposed a new mechanism for microvascular dysfunction, in which various atherosclerotic conditions and metabolic disorders lead to the conversion of endothelium-dependent relaxing mediators from NO to H_2_O_2_ (ceramide-induced reduction in mitochondrial telomerase activity has been shown to cause this conversion) [84,85]. As a consequence, the pathological levels of H_2_O_2_ act similar to a double-edged sword, which results in microvascular dysfunction and the development of coronary artery diseases [86]. Deficiency of eNOS caveolin-1 negative regulator or overexpression of eNOS will disrupt the physiological balance of EDH factors (e.g., NO, H_2_O_2_) in the microcirculation, which contributes to the overproduction of endothelial NO in mice with cardiovascular lesions [87]. Among the redox-regulated proteins, endothelial thioredoxin reductase 2 has been demonstrated to play a role in maintaining the healthy endothelial function [88], while peroxisome proliferator receptor-γ coactivator 1α is also considered to be a principal regulator of endothelial function, including the prevention of OS, inflammation, and atherosclerosis [89].

Hyperhomocysteinemia (HHcy) is an independent risk factor for cerebrovascular, cardiovascular, and peripheral arterial disease [90]. HHcy-related ED is a complex mechanism, whereby HHcy increases ROS production by a variety of mechanisms (e.g., HHcy autoxidation), which induces OS and an inflammatory state triggering the secondary cardiovascular disease, including atherosclerosis, hypertension, and neurodegeneration [91]. HHcy could cause an imbalance between antioxidant and oxidative enzymes by inhibiting SOD or activating NADPH oxidase [92]. Furthermore, auto-oxidation of the free thiol group of HHcy will generate H_2_O_2_, ROS, HO•, and O_2_^•^, with which NO could react to compose ONOO-, thereby reducing the bioavailability of NO [93]. Both in vitro and in vivo experiments have reported that HHcy can block GPx activity and HO-1, which promote ROS accumulation and exacerbate damage to ECs [94,95]. In addition, high plasma levels of HHcy induced apoptosis in ECs, due to endoplasmic reticulum stress, and increased ROS production and HHcy–thiolactone production [96].

Cells perceive and respond to the mechanical properties and cues of the microenvironment [96,97]. For example, this “mechanical sensing” allows vascular ECs to change the morphology, function, and gene expression in response to shear stress [98,99]. Mechanical stress, such as periodic tensile or shear stress, also stimulates NADPH oxidase activity in ECs [100,101]. However, elevated blood pressure (BP) itself can trigger damage to endothelial function and vascular remodeling [102]. The chronic presence of high BP was found to elicit increased arterial superoxide production by activating directly a protein kinase (PK) C-dependent NADPH oxidase pathway but also, in part, via activation of the local renin–angiotensin system [103].

## 5. The Role of Endothelial Dysfunction in Neurodegenerative Diseases in Central Nervous System

ED plays a key role in the pathogenesis of insufficient blood supply to various organs and tissues, including the brain [104], heart [105], and eyes [106], resulting in increased blood pressure [107,108] and insulin resistance [109]. Many neurodegenerative diseases have been shown to have pathological changes in ED, such as atherosclerosis, cancer, sepsis, Alzheimer’s disease (AD), and multiple sclerosis [110]. ED, at the junction of blood vessels and peripheral nerves, is involved in a variety of diseases through different mechanisms, including decreased levels of NO, increased expression of pro-inflammatory factors, and changes in endothelial cell permeability [111]. ECs in the brain form the blood–brain barrier (BBB), which strictly regulates the solute exchange between the vascular lumen and brain parenchyma. The destruction of BBB leads to the accumulation of blood-derived neurotoxic proteins (such as fibrinogen, thrombin, hemoglobin, ferritin, and free iron) in the brain parenchyma, which leads to neurodegenerative changes [112,113]. The patients and animal models of AD have demonstrated that the decrease in BBB tension is due to Aβ accumulation, leading to dysfunction of ECs and decreased expression of tight junction proteins in brain ECs [114,115,116,117,118]. Recently, some studies have shown that brain microvascular ECs produce amyloid-β peptide, which is perhaps a potential endothelium-dependent pathway that participates in Aβ deposition [119]. A potential pathway is the activation of proinflammatory cytokines induced by stress, which have been shown to induce the expression of adhesion molecules on the surface of vascular ECs [120].

## 6. The Pivotal Role of Endothelium Underlying Pathophysiology of Neurodegenerative Diseases in Retina

**Figure 2:**
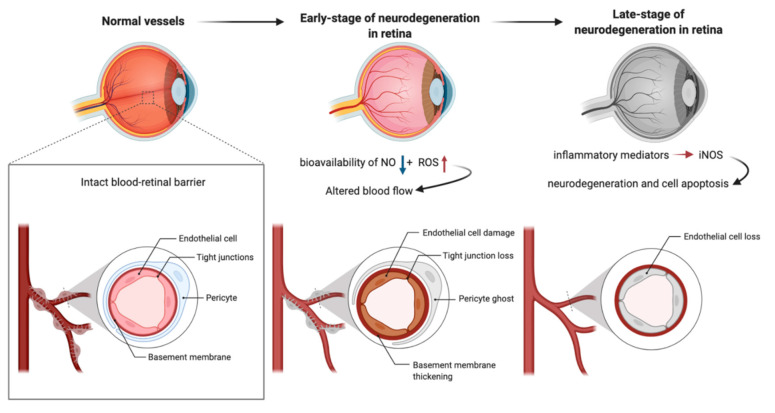
Endothelium and neurodegenerative diseases in retina.

The central retinal artery provides nutrition and oxygen for neurons in the retina [121]. ECs are arranged in the lumen of microvessels as physical barriers between blood and surrounding tissues and play key roles in regulating retinal homeostasis [122]. Pericytes wrap around retinal capillaries and regulate the function of ECs [123]. In addition to maintaining the structural support of the vascular wall, peripheral cell coverage also regulates the expression of tight junction protein in adjacent ECs [123]. The basement membrane separates pericytes from ECs; however, the pores in this membrane matrix allow the formation of intercellular junctions between pericytes and ECs [124,125]. Outer blood–retinal barrier (oBRB) composed of retinal pigment epithelium (RPE) and internal BRB (IBRB) consisting of ECs protect retinal nerve cells from harmful molecules in circulation [126]. Tight junctions between adjacent RPE cells and ECs play regulatory roles in the strict control of fluid and solute crossing the blood–retinal barrier, which prevents toxic molecules and plasma components from entering the retina [126]. In a recently conducted meta-analysis investigating levels of oxidative stress markers and antioxidants in patients suffering from conical cornea for the first time, the authors reviewed 36 articles, published until 1 June 2020, with a total of 1328 conical cornea patients and 1208 healthy controls. Compared with healthy controls, patients with keratitis presented with an overall increase in oxidative stress markers (standard mean deviation (SMD) = 0.94; 95% confidence interval (95% CI) 0.55–1.33), accompanied by a decrease in antioxidants (−0.63, −0.89 to −0.36), which resulted in a significant decrease in total antioxidant capacity/status (−1.65, −2.88 to −0.43). Moreover, oxidative stress markers were found to increase in stromal cells, while antioxidants were reduced in endothelial cells [127]. In order to achieve normal visual function, highly coordinated activities of neurons, glial cells, microglia, and microvessels are essential. Ocular blood flow is adjusted by NO secreted by ECs and efferent nitroergic neurons. ED impairs ocular hemodynamics by reducing the bioavailability of NO and increasing the production of ROS. On the other hand, the production of NO by inducible nitric oxide synthase (iNOS) under the action of inflammatory mediators leads to neurodegeneration and cell apoptosis, which initiate serious eye diseases (Figure 2).

The endothelium-derived NO possesses multiple complex physiological functions, such as inducing vasodilation, reducing vascular resistance, decreasing blood pressure, inhibiting platelet aggregation and adhesion, inhibiting leukocyte adhesion and migration, reducing smooth muscle hyperplasia, and preventing atherosclerosis. Shear stress, vascular endothelial growth factor (VEGF), insulin, or bradykinin can induce the phosphorylation of eNOS through phosphatidylinositol-3 (PI3) kinase and downstream serine/threonine–protein kinase Akt (protein kinase B), which leads to the enhancement of NO synthesis [128,129,130]. In the bovine retina, shear stress stimulates NO release from bovine retinal ECs monolayer [131]. VEGF increases retinal endothelial cell permeability through an eNOS dependent caveolae cell transport mechanism [132] or directly increases NO release from bovine choroidal ECs [133]. Michelson et al. [134] demonstrated that NOS-dependent vascular tension in retinal arteries and capillaries of patients with early hypertension is impaired. iNOS elicited a large amount of NO production is related to inflammatory response, as well as increased OS caused by hyperglycemia and ED, which is involved in the pathological changes in the retina and associated tissues in glaucoma and diabetic retinopathy. In a double-blind crossover study of healthy male subjects, Dallinger et al. [135] revealed that infusion of L-arginine (rather than D-arginine) increased choroidal pulsatile blood flow, mean ophthalmic artery velocity, and renal plasma flow, and that insulin infusion enhances vasodilation of L-arginine. They concluded that this stereo-specific vasodilation may be due to the promotion of membrane transport of L-arginine, which enhances the formation of intracellular NO or increases the bioavailability of NO. Intravenous injection of L-arginine can reduce mean arterial pressure and increase choroidal blood flow and retinal vein blood flow in healthy volunteers [136].

In healthy conditions, most ECs remain static and participate in the maintenance of barrier function and tissue homeostasis [137]. However, hyperglycemia induces OS in retinal endothelial cells (RECs), which further activates and interferes with a variety of metabolic pathways, leading to the self-perpetuating cycle of harmful OS and promoting the development of DR [138,139]; the ultimate consequence is to reduce the integrity of the blood–retinal barrier [140]. The OS of REC induced by hyperglycemia results from mitochondrial dysfunction [141]. Under the condition of hyperglycemia, the excessive production of mitochondrial ROS leads to mitochondrial disruption and dysfunction, which changes REC metabolism into hyperglycemia and reduces the ability of REC to maintain DR barrier function and tissue homeostasis [142,143]. Delles et al. [144] propose that retinal vascular endothelial function is impaired in young patients with early primary hypertension, but angiotensin AT1 receptor blockade can improve its function. However, several studies have shown that vitamins could affect the process of common eye diseases. Vitamins A, B9, C, and E are well-known antioxidants that prevent age-related eye diseases, for instance, cataracts and age-related macular degeneration [145,146]. A meta-analysis reported that dietary intake of vitamins A and C was beneficially correlated with OAG; however, the results of studies on the levels of vitamins in blood failed to provide a definitive relationship with OAG [147].

## 7. Schlemm’s Canal and Glaucoma

Elevated intraocular pressure (IOP) is a major risk factor of retinal ganglion cell degeneration in glaucoma, which is caused by increased resistance to the outflow of aqueous humor (AH). Trabecular meshwork (TM) and Schlemm’s canal (SC) endothelial are key regulators of outflow resistance. In glaucomatous eyes, the TM–SC pathway is damaged by accumulated oxidative stress arising from the microenvironment, vascular dysregulation, and aging [148]. As the final filtration barrier of the AH outflow pathway, stiffness of SC endothelia are crucial to the regulation of aqueous humor outflow resistance and control of IOP [149]. Schlemm’s canal endothelial cells share morphological characteristics and cell marker expression with both lymphatic and venous endothelial cells [150]. Similar to that of other ECs, Schlemm’s canal ECs also respond to changes in substrate stiffness or architecture. In particular, glaucomatous SC cells have an exaggerated and possibly pathological response to increased substrate stiffness, compared with normal SC cells [149]. They alter their expression of genes implicated in outflow obstruction and glaucoma in response to changes in substrate stiffness, particularly connective tissue growth factor (CTGF), an agent which has been shown to cause ocular hypertension and glaucomatous optic neuropathy in mice [151].

Thus, targeting SC cell stiffness seems to be a promising antiglaucoma therapy to decrease outflow resistance at the location responsible for regulating the majority of outflow resistance. Drugs targeting Rho-kinase inhibition, actin depolymerization, and nitric oxide were recently introduced to reduce SC cell stiffness and reduce IOP [152,153,154,155].

## 8. Conclusions

It is widely accepted that endothelial dysfunction plays a crucial role in the development of neurodegeneration. There is a potential pathological link between oxidative stress, endothelial function, and neurodegenerative diseases of the retina. A better understanding of the roles of endothelial function may provide new treatments for retinal neurodegenerative diseases.
